# Development of the Brazilian Anti Schistosomiasis Vaccine Based on the Recombinant Fatty Acid Binding Protein Sm14 Plus GLA-SE Adjuvant

**DOI:** 10.3389/fimmu.2015.00218

**Published:** 2015-05-12

**Authors:** Miriam Tendler, Marilia Almeida, Andrew Simpson

**Affiliations:** ^1^Oswaldo Cruz Institute, Oswaldo Cruz Foundation, Rio de Janeiro, Rio de Janeiro, Brazil; ^2^Orygen Biotecnologia, São Paulo, São Paulo, Brazil

**Keywords:** vaccine, schistosomiasis, Sm14, disease of poverty, FABP, Brazil

## Abstract

Data herein reported and discussed refer to vaccination with the recombinant fatty acid binding protein (FABP) family member of the schistosomes, called Sm14. This antigen was discovered and developed under a Brazilian platform led by the Oswaldo Cruz Foundation, from the Health Ministry in Brazil, and was assessed for safety and immunogenicity in healthy volunteers. This paper reviews past and recent outcomes of developmental phases of the Sm14-based anti schistosomiasis vaccine addressed to, ultimately, impact transmission of the second most prevalent parasitic endemic disease worldwide.

## Introduction

Schistosomiasis is considered by the World Health Organization to be 1 of 17 neglected tropical diseases (1). With 800 million people at risk, and 200 million infected in 74 countries, schistosomiasis is the second most prevalent human, parasitic disease in the world after malaria. Some 7.1 million people are infected with *Schistosoma mansoni* in the Americas, of whom 95% are in Brazil (1). It is estimated that 25 million people are exposed to the risk of schistosomiasis in the Americas (2). The WHO estimated that the morbidity of schistosomiasis resulted in the annual loss of 1.7 million disability-adjusted life years (DALYs), while mortality was estimated to be 41,000 deaths per year (3). Control measures aim to reduce morbidity through treatment with praziquantel, improved sewerage, access to potable water, and snail control (1–4). Vaccination, even if not 100% effective, could contribute to the long-term reduction of egg-excretion from the host, and thus controls transmission. An effective vaccine would also contribute to a positive trade-off regarding the aggressive inflammatory response that has been observed following interrupted chemotherapy in children living in high-transmission areas (5–7). The underlying reason for this “rebound morbidity” is unclear, but is thought to be due to an interruption of the natural down-regulating process of specific immunological mechanisms typical for this disease. This outcome results from the typically high-level re-infection after chemotherapy and is a direct result of chemotherapy being primarily directed against morbidity and less against transmission of the disease. This effect needs to be taken seriously, as the observed aggravated gross symptoms reflect long-term pathology, which is difficult to remedy (8).

There have been initiatives in several countries to develop a vaccine against schistosomiasis. The Brazilian Sm14-based anti schistosomiasis vaccine is the sole technology that emerged from an endemic country, and that is at an advanced stage of development toward a safe highly innovative product.

In this review, we present the evolution of the Sm14 anti-schistosome vaccine from the initial gene cloning to the results of the recently completed phase I clinical trials.

## Demonstration That Adult Schistosome Saline Extracts Contain Protective Antigens

The experimental background for the development of anti-schistosome vaccines lies with the use of animal models of infection that showed that an initial parasite infection resulted in partial immunity against re-infection (9–11). Levels of resistance achievable in laboratory models ranged from 60% in mice up to 90% in rabbits. In an attempt to establish whether vaccine development was feasible, extracts of adult parasites were utilized to immunize experimental hosts to investigate whether such antigen preparations also possessed the capacity to protect against infections. In two independent lines of investigation, it was demonstrated that simple saline extracts of live adult worms, which are enriched in surface associated molecules, were indeed capable of inducing protection comparable to that achieved with live infection (12–14).

## Adult Worm Antigen Gene Cloning

Once gene-cloning technology became incorporated into the vaccine research field, the genes for a number of major antigens released by adult schistosomes briefly cultured in saline were cloned and sequenced (15–17). One of these proved to be a fatty acid binding protein (FABP) termed Sm14, which subsequently became the basis of the experimental vaccine herein discussed. The protein derived from the cloned gene exhibited significant homologies with a family of related polypeptides, which bind hydrophobic ligands, and purified recombinant protein exhibited an affinity to fatty acids. Antibodies to the purified protein were shown to bind to tubercles, which are structures located on the dorsal surface of adult male schistosome and known to contain lipids (17). In addition, the protein was localized to the muscle layers as well as in the body of the parasite. As the schistosome cannot synthesize fatty acids *de novo*, and is dependent on the uptake of lipids from serum, the available data supported a role for Sm14 in the transport of fatty acids. Following transfer of the Sm14 gene to a high level expression vector, subsequent experiments demonstrated that the recombinant rSm14 was able to protect outbred Swiss mice by up to 66% and New Zealand White rabbits by up to 89% against challenge with *S. mansoni* cercariae. It was thus demonstrated that rSm14 could provide the basis of an anti-schistosome vaccine (18).

## Vaccine Development

**Figure 1:**
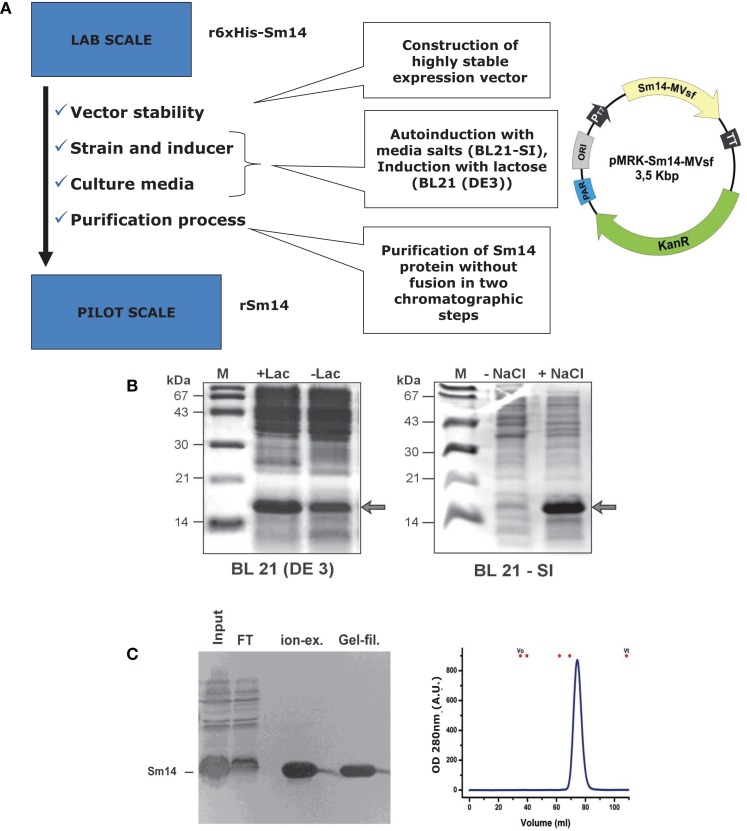
**Expression of Sm14 in *Escherichia coli***. **(A)** Steps taken to improve the production process of Sm14 in *E. coli*. **(B)** Induction/expression systems with lactose (left) and salt (right). **(C)** Final purification steps involving ion exchange chromatography or gel filtration.

**Figure 2:**
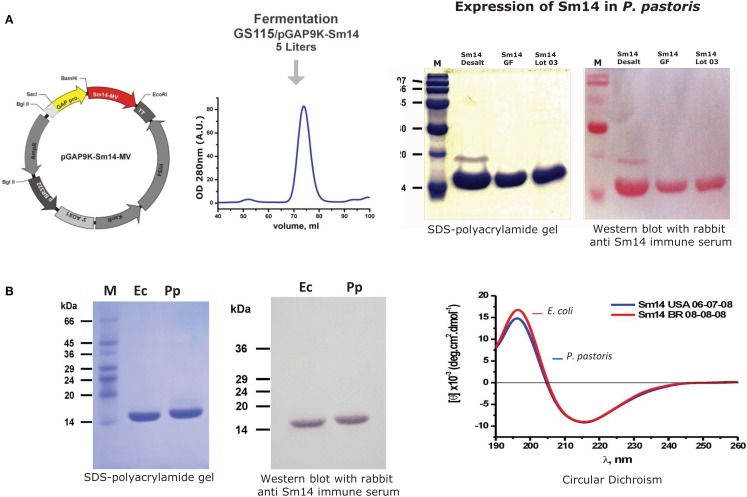
**Expression of Sm14 in *Pichia pastoris***. **(A)** Purification and detection of Sm14 expressed in *P. pastoris*. **(B)** Comparison between Sm14 protein batches purified using both platforms; *E. coli* (Ec) versus *P. pastoris* (Pp).

The Sm14 project has been mostly funded by public funds at Oswaldo Cruz Institute/FIOCRUZ, belonging to the Brazilian Ministry of Health. Since the early stage of the development, there was a critical concern to reduce the cost of production at its lowest level and to ensure the use of non-proprietary components in the production process in order to get a low final price for the vaccine. The strategy adopted to reduce the cost of production of the human Sm14 vaccine involved the following steps:

- Scaling up steps of production process: this started in 2003 with the primary target being the investigation of Sm14 stability. New constructs were developed with highly stable novel molecular design (19), beginning thus to pave the way for the ultimate goal of achieving large-scale production of highly purified vaccine at both high yield and low cost.
- Replacement of more expensive reagents seeking a royalty and proprietary free route of components: as presented in Figures 1 and 2, two substitutions were successfully achieved, which were the replacement of IPTG for lactose or salt (NaCl), for the steps of induction of protein expression in culture. Expression vectors were also constructed to avoid commercial ones. Furthermore, the Sm14 vaccine is based only on two highly purified and well-characterized components: the protein itself and the glucopyranosyl lipid adjuvant stable emulsion (GLA-SE) adjuvant produced and supplied by the Infectious Disease Research Institute (IDRI, Seattle). Our partner, IDRI, is a non-profit organization fully committed to the support of the development of technologies for the control of the so-called neglected diseases.
- Production process of the protein in large-scale is presently in place at Ourofino, the partner for the veterinary vaccine. There is currently pilot scale production of the vaccine in 5 L fermentor, which is being scaled-up to 50 and 100 L fermentors, with final cost already estimated to be approximately US $1,00) for one 50 μg dose.

A series of modifications of the prototypic experimental Sm14 vaccine, which consisted of a fusion protein presented with RIBI adjuvants (oil-in-water emulsions derived from bacterial and mycobacterial cell wall components), were undertaken to gradually convert the original laboratory-bench protein into a clinical product. First, it was demonstrated that rSm14 could be produced in a non-fused form while retaining its protective immunogenicity (20). A genomic polymorphism was identified in the Sm14 gene whereby the conserved methionine at position 20 is polymorphic, being exchangeable with threonine (M20T) (21). Both forms were found to be protectively immunogenic to adopt the same three dimensional structure in solution and to be functional in that they were able to bind fatty acids. The M20 isoform was found to exhibit superior stability, however, and was adopted for further vaccine development. A variety of approaches to vaccine formulation were explored in which it was demonstrated that short peptides derived from the C-terminal of sm14 were capable of conferring equivalent levels of protective immunity to experimental animals as intact rSm14 (22). rSm14 was found to be protective when presented in a live vaccine form within recombinant *Mycobacterium bovis* BCG (23, 24), and that rSm14 expressed as a fusion with tetanus toxin fragment C induced immunoprotection against schistosomiasis in mice (25). Last, a modified version of the protein with improved stability due to the avoidance of dimerization and subsequent aggregation was engineered by Cys62 replacement. The latter version was adopted as the lead compound for vaccine development (19).

The final steps toward a clinically applicable formulation of rSm14 were taken by developing a *Pichia pastoris* based expression system for the protein (26), and further process development resulted in modifications that avoided any proprietary genetic structures or media requirements enabling low-cost manufacturing. In addition, the synthetic adjuvant GLA-SE was selected for incorporation into the final product, which has now been utilized in Phase I clinical trials since this adjuvant enhances the Th-1 type responses such as gamma-interferon production that have been identified as representing the basis of Sm-14 mediated protective immunity both in human patients and animal models (see below) (27). The good manufacturing practice (GMP) production of rSm14 for clinical trials was undertaken at the LICR protein production facility at Cornell University in Ithaca, NY.

## Immune Response to Sm14

The collaborative research group based at the *Fundação Oswaldo Cruz* has focused exclusively on the stepwise development of schistosomiasis vaccine guided solely by molecular considerations together with assays of protective immunity in animal models. On the other hand, other groups have investigated the nature of the immune response elicited by the antigen particularly in the context of natural human infection and the development of resistance to infection. As a first step, it was demonstrated that the sera of schistosomasis patients contain significant amounts of IgG1 and IgG3 subclass antibodies, whereas low levels of IgM, IgA, or IgE were measurable (28, 29). Specifically, the cellular immune responses to rSm14 were examined in chronic, treated patients and uninfected individuals living in an endemic area for schistosomiasis. Lymphocyte proliferative responses to rSm14 were detectable in the peripheral blood mononuclear cells of all groups studied with the highest proliferation index to rSm14 being detected in uninfected endemic normal (EN) individuals who are naturally resistant to schistosomiasis (28). This result provides direct evidence that the immune response to Sm14 may contribute to protective immunity in man. Moreover, it was determined that lymphocyte proliferation in the uninfected group was dependent on IFN-gamma suggesting that the Th1 might be associated with resistance to infection. Furthermore, analyses in mice suggested that the same immune effector mechanism may be responsible for the protective immunity stimulated by rSm14 vaccination, i.e., that the schistosome vaccine based on Sm14 may reproduce naturally occurring protective immunity in man (30). Interestingly, the strong parallel relationship between naturally occurring immunity and rSm14 vaccination could be extrapolated to the molecular level. T-cell epitopes were identified within the molecule that are recognized by T-cells producing gamma interferon from resistant individuals. Furthermore, the peptide epitopes from Sm14, but not from another schistosome antigen (paramyosin), stimulated protective immunity and gamma-interferon producing T-cells in vaccinated mice (31, 32).

## Phase I Clinical Trials

Following approval by the local Ethics Committee and the Brazilian Regulatory Agency, ANVISA, two separate Phase I clinical trials of the rSm14/GLA-SE vaccine have been undertaken with healthy volunteers. The first trial involved 20 males and the second involved 10 females (www.clinicaltrials.gov) (Number NCT01154049). These trials demonstrated that the vaccine is safe and immunogenic. The vaccine was administered intramuscularly in three 0.5 mL doses, each containing 50 μg of both rSm14 and GLA-SE. The second dose was administered 8 weeks after the first one, while the third dose was given 1–2 months later. There were no serious adverse events reported with the only side effects being mild local pain at the site of vaccination in some individuals. With the support of Infectious Disease Research (IDRI, Seattle, USA) clinical trial team, cells and sera from Brazilian volunteers were shipped to Seattle and extensively screened for the immune response generated by vaccination with Sm14 + GLA-SE toward the identification of the immunological signature of human immunization. Vaccination stimulated anti-Sm14 IgG antibodies as well as a Th1 T-cell response, resulting in gamma-interferon production in the vaccinated individuals. (manuscript submitted to Vaccine).

## rSm14 as a Multi-Specific Anti-Helminth Vaccine

It has long been known that there is cross reactive protective immunity between the animal parasite *Fasciola hepatica* and schistosomes. Analysis of the molecular basis of this protective response identified a cross reactive antigen present in *F. hepatica*, termed Fh15 (33). The cloning and sequencing of Sm14 revealed this to be the corresponding protein in *S. mansoni*. Molecular models showed that Fh15 and Sm14 adopt the same basic three-dimensional structure, consisting of a barrel-shaped molecule, and also identified shared discontinuous epitopes principally derived from amino acids in the C-terminal portions of the molecules. Moreover, rSm14 provided complete protection against challenge with *F. hepatica* metacercariae in a mouse model, suggesting that it may be possible to produce a single vaccine that would be effective against at least two parasites, *F. hepatica* and *S. mansoni*, of veterinary and human importance, respectively (18). Further analysis confirmed these initial findings and also demonstrated that vaccination with rSm14 can protect the natural host of *F. hepatica*, the sheep, against experimental parasite challenge resulting in complete abolition of liver pathology (34). In independent experiments undertaken in Spain, protection against *fasciola* infection was later also achieved in goats immunized with rSm14, where again significantly reduced liver damage was recorded (35). Several groups around the world have been undertaking studies to evaluate the potential of FABPs homologous to Sm14 derived from various organisms as vaccines against a number of different helminth diseases. Those include diseases caused by *F. hepatica* (18, 36–41), *S. mansoni* (18, 42), *Schistosoma japonicum* (43, 44)*, Echinoccus granulosus* (45), and *Clonorchis sinensis* (46) in both experimental and natural veterinary hosts. The published reports from these groups provide a robust dataset, indicating the widespread potential efficacy of vaccines based on Sm14. Although experimental work has been undertaken with the FABPs from many parasites, only Sm14 has reached the stage of GMP production and clinical trials.

## Future Perspectives

To conclude all pre-clinical stages, Sm14 project has overcome bottlenecks of a vaccine development, scaled-up the production, formulated the product, and fulfilled all regulatory requirements to start clinical study phase. The national regulatory authority (ANVISA) approved the results of phase 1 clinical studies, and Fiocruz has licensed Sm14 technology to a Brazilian company, Ourofino, for final development and commercialization of Sm14 vaccine for use in cattle herds against *fasciola*.

Field based immunogenicity and safety phase 2 trials of the Brazilian Sm14 + GLA-SE anti schistosomiasis vaccine are planned to start in 2015 in endemic areas (Brazil and Africa).

## Conflict of Interest Statement

The authors declare that the research was conducted in the absence of any commercial or financial relationships that could be construed as a potential conflict of interest.
